# Methylation and expression of PTPN22 in esophageal squamous cell carcinoma

**DOI:** 10.18632/oncotarget.11581

**Published:** 2016-08-24

**Authors:** Jiaying Deng, Junhua Zhang, Chunyu Wang, Qing Wei, Daizhan Zhou, Kuaile Zhao

**Affiliations:** ^1^ Department of Radiation Oncology, Fudan University Shanghai Cancer Center, Shanghai 200032, China; ^2^ Department of Oncology, Shanghai Medical College, Fudan University, Shanghai 200032, China; ^3^ Department of Pathology, Tenth People's Hospital of Tongji University, Shanghai 200072, China; ^4^ Bio-X Center, Key Laboratory for the Genetics of Developmental and Neuropsychiatric Disorders (Ministry of Education), Shanghai Jiao Tong University, Shanghai 200030, China

**Keywords:** PTPN22, ESCC, methylation, expression, prognosis

## Abstract

Esophageal squamous cell carcinoma (ESCC) is a fatal disease contributed by both genetic and epigenetic factors. The epigenetic alteration of protein tyrosine phosphatase non-receptor type 22 (PTPN22) and its clinical significance in ESCC were still not yet clarified. A quantitative methylation study of PTPN22 and its expression were conducted in 121 and 31 paired tumor and adjacent normal tissue (ANT), respectively. Moreover, the association between PTPN22 methylation and clinicopathological parameters was evaluated. We found that the methylation level of PTPN22 was significantly elevated in tumor tissues (66.3%) relative to ANT (62.1%) (*p*=0.005). The methylation level of non-smoking ANT (59.1%) was significant lower than smoking ESCC tissue (65.8%) (*p*=0.03); similarly, the methylation levels in ANT with no lymph node invasion (57.6%) were significant lower than tumor tissues with lymph node invasion (67.5%) (*p*=0.001). PTPN22 expression in ESCC was lower than normal tissues, however the difference was not statistically significant (*p*=0.55). Lower expression was more frequently occurred in N_1-3_ and III stage patients, while higher expression was more likely to occur in N_0_ and I-II stage patients. Lower expression of PTPN22 was associated with poor overall survival (*p*=0.04). Taken together, PTPN22 was hypermethylationed in ESCC. Hypermethylation was associated with lymph node invasion. The PTPN22 expression may act as a prognostic biomarker to identify patients at risk of high grade.

## INTRODUCTION

Esophageal squamous cell carcinoma (ESCC) is one of the most prevalent cancers in African and Asian countries, accounting for >90% of all esophageal carcinomas in the worldwide [1]. Approximately 482,300 new cases of esophageal cancer are diagnosed annually, and the disease is responsible for approximately 406,800 deaths each year [2]. The disease is often advanced at presentation and has a poor prognosis despite the use of multidisciplinary therapy. The biological and pathological features of ESCC have not been well demonstrated, although multiple genetic and epigenetic changes have been detected in ESCC [3, 4]. Therefore, additional understanding of the molecular mechanisms of esophageal cancer is urgently needed to devise more effective treatment.

DNA methylation is one of the most common epigenetic modifications in mammalian genomes [5]. Aberrant methylation of gene promoter subsequently results in the inactivation of gene expression. In particular, hypermethylation of cytosine-guanine dinucleotide (CpG) islands in promoter regions has been strongly implicated in the onset and progression of cancer [6]. Aberrant methylation of the gene promoter has become widely recognized as a mechanism of gene inactivation in cancer [7].

The protein tyrosine phosphatase non-receptor type 22 (PTPN22) gene is located on chromosome 1p13.3–13.1 and participates in epithelial adhesion. In chronic lymphocytic leukemia (CLL), a study showed that overexpression of PTPN22 significantly inhibits antigen-induced apoptosis of primary CLL cells by blocking B-cell receptor (BCR) signaling pathways that negatively regulate lymphocyte survival. More importantly, this finding indicates that PTPN22 positively regulates antiapoptotic AKT kinase, providing a powerful survival signal to antigen-stimulated CLL cells [8]. Several large-scale comprehensive analyses, which were conducted using high-throughput sequencing technology, have revealed that the epidermal growth factor receptor (EGFR) signaling pathway and related downstream pathways, such as the PI3K/AKT signaling pathway, are considered to be involved in the development of ESCC [9–11]. Additionally, the TCGA database showed that there was a significant difference in the methylation level of PTPN22 between tumor tissues and normal tissues. However, despite the potential importance of the PTPN22 gene in carcinogenesis, there have been very few reports concerning ESCC. No study has been conducted regarding the expression and methylation of PTPN22 in ESCC. In the present study, we attempted to ascertain the methylation level and expression of PTPN22 in ESCC and the correlation between the PTPN22 methylation change and a series of clinicopathological parameters in a large sample of ESCC patients to elucidate the role of PTPN22 in the pathogenesis of ESCC and its potential role as a prognostic marker.

## RESULTS

### Methylation analysis of PTPN22

**Figure 1:**
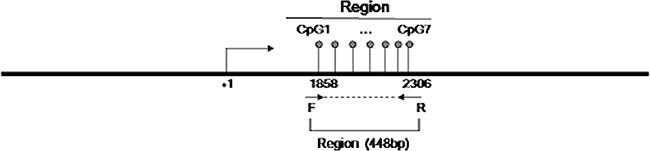
Schematic diagram of the CpG sites in the selected region The CpG sites are depicted by lollipop markers. The binding sites for the forward and reverse primers are depicted as arrows below the diagram.

**Figure 2:**
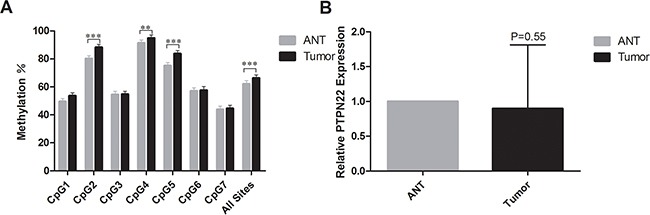
**A.** The mean methylation level for each CpG site in PTPN22. ***represents *p*<0.001, **represents *p*<0.01, and *represents *p*<0.05; **B.** The mRNA expression level of PTPN22. *represents *p*<0.05.

Using Vector NTI Advance 11 (Invitrogen), we compared the CpG density in different regions of the promoter and chose to analyze the region chr1:114,358,291-114,358,739 [GRCh37/hg19]. The selected region contains elevated CpG content and is easy to amplify. The amplicon was designed and included seven CpG sites (Figure 1). All seven CpG sites (CpG1, CpG2, CpG3, CpG4, CpG5, CpG6, and CpG7) were successfully detected using the quantitative Sequenom method (Table 1). The threshold value for methylation detection was 10%. The methylation level of the CpG sites in the selected region was subjected to paired analyses; three of the seven CpG sites (CpG2, CpG4, CpG5) exhibited significant differences between ANT and tumor tissue (80.2% vs. 88.3%, 91.4% vs. 94.9%, and 75.2% vs. 83.8%, respectively). All three of these CpG sites were significantly hypermethylated in ESCC tissues (Figure 2A). The mean methylation level of all CpG sites was 62.1% in ANT and 66.3% in tumor tissue. The difference was statistically significant (*p*=0.005, Table 1).

**Table 1:** Methylation level (%) of the detected sites in the selected region

CpGs	Group	Mean	ΔMean	*P*
CpG1	Normal	49.5	4.2	0.127
	Tumor	53.7		
CpG2	Normal	80.2	8.1	<0.0001
	Tumor	88.3		
CpG3	Normal	54.5	0.3	0.231
	Tumor	54.8		
CpG4	Normal	91.4	3.5	0.007
	Tumor	94.9		
CpG5	Normal	75.2	8.6	0.0002
	Tumor	83.8		
CpG6	Normal	57.1	0.5	0.798
	Tumor	57.6		
CpG7	Normal	43.9	0.7	0.836
	Tumor	44.6		
All Sites	Normal	62.1	4.2	0.005
	Tumor	66.3		

ΔMean: the difference of mean methylation level between tumor and adjacent normal tissue

### Relative PTPN22 expression level in ESCC samples by real-time PCR

**Figure 3:**
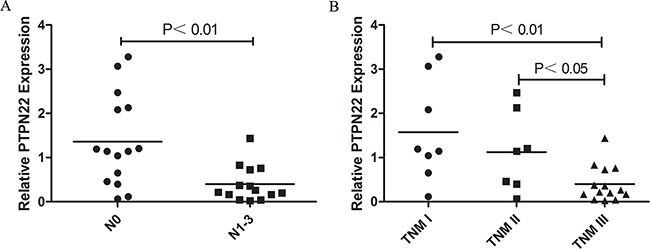
**A.** Correlation of PTPN22 expression and lymph node invasion; **B.** Correlation of PTPN22 expression and TNM stage.

**Figure 4:**
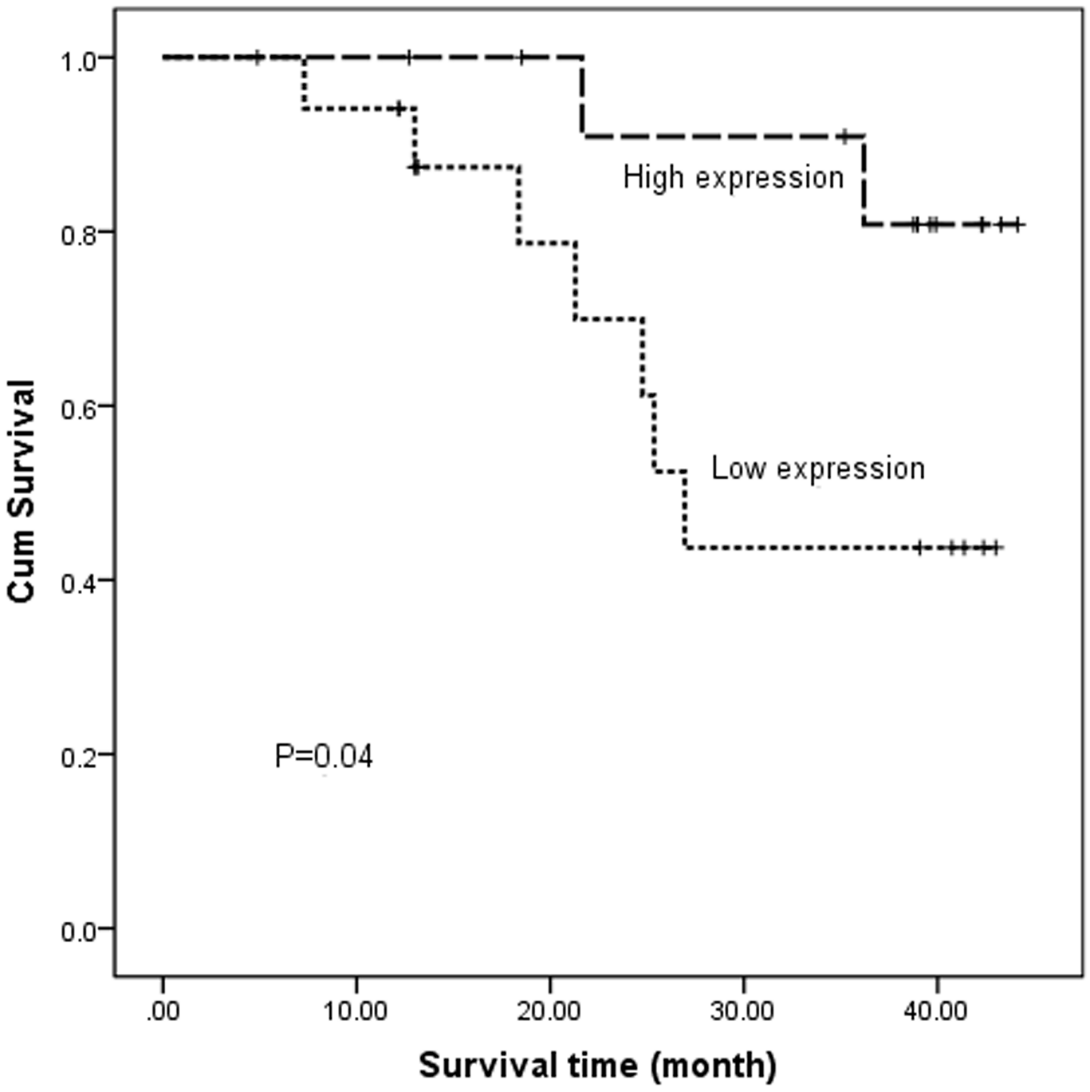
Kaplan–Meier estimates of overall survival for ESCC patients according to different PTPN22 expression levels

The relative PTPN22 expression level was quantified in 43 paired tumor tissues and ANT. All samples were selected from the same cohort with methylation detected and run in triplicate to capture intra-assay variability. Then 31 paired samples were finally included to be analyzed. Lower expression was observed in 18 tumor samples, and higher expression was observed in 13 samples. The relative PTPN22 expression level was reduced in the tumor tissue compared with that in the corresponding ANT, but the difference was not statistically significant (ANT vs. tumor: 1 vs. 0.9, respectively; *p*=0.55) (Figure 2B). Linear regression analysis showed that lymph node metastasis and TNM stage were inversely associated with the expression level. Lower expression was more frequently observed in N_1-3_ and III stage patients, while higher expression was more likely to occur in N_0_ and I-II stage patients (Figure 3). The results were consistent with the reduced expression level in tumor tissue. Furthermore, overall survival was better in patients with lower PTPN22 expression than in patients with a higher expression level (Figure 4, *p*=0.04).

### Correlation of the PTPN22 methylation change and clinicopathological data

We analyzed the correlation between the methylation change (tumor/ANT) and multiple clinicopathological parameters. Smoking and N stage were found to be associated with the methylation change between the tumor and ANT samples (*p<*0.05) (Table 2). A distinct methylation change existed in non-smoking and N_0_ patients compared with smoking and N_1-3_ patients (8.3% vs. 2.1%, 7.5% vs. 1.4%, respectively). Additionally, the methylation level in the ANT of non-smokers was significantly lower than that in the ESCC tissue of smokers (59.1% vs. 65.8%, *p*=0.03); similarly, the methylation levels in ANT with no lymph node invasion were significant lower than those in tumor tissues with lymph node invasion (N_0_ 57.6% vs. N_1-3_ 67.5%, *p*=0.001). For other analyzed elements, including age, alcohol habit, nerve invasion, vessel invasion and TNM stage, there was no significant association with the methylation change in ESCC (Table 2).

**Table 2:** Association between methylation difference (%) and clinicopathological parameters

Parameters	NO.	Normal	Tumor	ΔMean	*P*
Gender					0.66
Male	111	61.4	65.7	4.3	
Female	10	70.6	72.7	2.1	
Age					0.53
<58	52	61.6	66.8	5.2	
≥58	69	62.6	66.0	3.4	
Alcohol habit					0.43
No	99	59.8	65.1	5.3	
Yes	86	65.1	67.9	2.8	
Smoking					0.05
No	41	59.1	67.4	8.3	
Yes	80	63.7	65.8	2.1	
Nerve invasion					0.63
No	95	62.4	66.9	4.5	
Yes	26	61.2	64.2	3.0	
Vessels invasion					0.86
No	102	62.5	66.5	4.0	
Yes	19	60.4	65.5	5.1	
T stage					0.88
T1-2	27	59.1	63.0	3.9	
T3	94	63.0	67.3	4.3	
N stage					
N0	57	57.6	65.1	7.5	0.04
N1-3	64	66.1	67.5	1.4	
TNM stage					0.13
I-II	61	58.6	65.1	6.5	
III	60	65.7	67.6	1.9	
Differentiation					0.46
Well	13	65.8	64.9	−0.9	
Moderate	68	61.1	66.5	5.4	
Poor	40	62.7	66.6	3.9	
Lesion location					0.15
Upper thoracic	44	61.7	67.9	6.2	
Middle thoracic	46	63.6	64.1	0.5	
Low thoracic	31	60.5	67.4	6.9	

### Association of the PTPN22 methylation level with overall survival and progression-free survival

To determine whether a correlation exists between PTPN22 methylation and patient survival, we analyzed five-year clinical follow-up data according to methylation levels. The PTPN22 methylation level was classified into two groups according to the cut-off value (70.3%), which was calculated as the mean of the normal samples (62.1%) to which we applied 0.5 SD (8.2%). A reduced methylation level in the tumor relative to the cut-off was defined as group 1. Similarly, an elevated methylation level in the tumor relative to the cut-off was defined as group 2. The 1-year, 3-year and 5-year rates of overall survival (OS) and progression-free survival (PFS) were analyzed using the Kaplan–Meier method (Table 3). No significant correlation was observed between methylation levels and prognosis (OS, *p*=0.554; PFS, *p*=0.614; respectively).

**Table 3:** Overall survival (OS) and progression-free survival (PFS) rates

		OS rate (%)				PFS rate (%)			
		1-year	3-year	5-year	*P*	1-year	3-year	5-year	*P*
Region	Group 1	83.5	58.1	42.2	0.554	73.6	46.5	31.6	0.614
	Group 2	79.7	49.5	40.4		71.4	32.4	30.6	

Group 1: decreased methylation level of tumor relative to the cut-off (70.3%)

Group 2: elevated methylation level of tumor relative to the cut-off (70.3%)

## DISCUSSION

In this study, we examined the methylation level of seven CpG sites in the selected region of PTPN22 (chr1:114,358,291-114,358,739 [GRCh37/hg19]) and observed that all seven sites exhibited hypermethylation in tumor tissue. The mean methylation level of all detected CpG sites was higher in ESCC. The selected region was not the conventional promoter region. The CpG site density in the promoter region was lower than that in the selected region in our analysis. There were only three or four CpG sites in the promoter region (Supplementary Figures 1, 3,000-5,200bp). According to the instructions of the SpectroCHIP® Arrays, quantification of at least four CpG sites was required for detection. In addition, the repeatability and consistency of the promoter region were poor because of rare CpG sites. Thus, combined with bioinformatics software, we selected the abovementioned region for analysis.

According to MATCH^TM^ public version 1.0 software (BIOBASE BIOLOGICAL DATABASES, http://www.gene-regulation.com/index2.html), Paired box gene 4 (PAX4) was found to be bound in the selected region. PAX4 is a transcriptional modulator located on chromosome 7q32. Its expression is dysregulated in various human cancers. Patients with high PAX4 expression levels demonstrated lower 5-year survival rates in HNSCC, gastric cancer and breast cancer. Multiple studies have suggested that PAX4 may act as a driver gene in multiple tumors [12, 13]. Studies have indicated that miR-144 and miR-451(miR-144/451) upregulation inhibits cancer cell migration and invasion. However, PAX4 upregulation decreased miR-144/451 levels. PAX4 promoted migration and invasion in human epithelial cancers by decreasing the miR-144/451expression level [14]. We hypothesize that PAX4 may act as a regulator of PTPN22 in the development of ESCC. The analysis of the expression of PAX4 in ESCC is warranted in a future investigation. *In vitro*, the expression of PAX4 should be introduced to analyze the effect of the demethylation of PTPN22 on the expression of PAX4. Future experiments will focus on investigating the relationship between PTPN22 and PAX4.

Hypermethylation of CpG islands in gene promoter regions always results in the down-regulation of a gene [15, 16]. In the current study, the relative expression of the PTPN22 gene in tumor tissue was lower than that in ANT, although the difference was not significant. Combined with the hypermethylation in tumor tissue, the current results suggest that aberrant PTPN22 methylation may suppress the expression of PTPN22 mRNA in esophageal cancer. Additionally, lower expression more frequently occurred in N_1-3_ and III stage patients than in N_0_ and I-II stage patients. Combined with the higher methylation level in N_1-3_ and III stage patients, these phenomena were also consistent with the notion that gene hypermethylation down-regulates gene expression. PTPN22 expression was associated with prognosis. Overall survival was poor in patients with lower PTPN22 expression than in the cases with a higher expression level. PTPN22 expression may act as a predictor of prognosis in ESCC.

To assess the clinical significance of PTPN22 methylation, we examined the association between PTPN22 methylation changes and the clinicopathological characteristics of esophageal cancer. The mean methylation level was elevated in the tumors of patients with lymph node invasion and a history of smoking relative to normal tissues (Table 2). This finding is consistent with the opinion that various environmental and lifestyle exposures, such as those related to tobacco, alcohol, and radiation, are implicated in the development of human cancers by inducing DNA methylation changes [17]. Studies have demonstrated that tobacco smoking is associated with aberrant gene methylation in several cancers [18, 19]. Additionally, some studies have suggested a trend toward a higher risk of advanced T stage (*p*=0.05) or lymph node metastasis (*p*=0.02) when more adverse gene methylation profiles are present [20–23]. Our results confirmed that smoking and lymph node metastasis are risk factors for carcinogenesis. The results revealed that the difference in the methylation level of tumor tissue and ANT was larger in non-smoking and N_0_ patients than in smoking and N_1-3_ patients.

The methylation level in well-differentiated tumors was lower than that in normal tissue (ΔMean=−0.9%; Table 2). The methylation change was significantly different from that in tumors with moderate or poor differentiation (ΔMean=5.4% or 3.9%, respectively; Table 2). The functional consequences of DNA methylation changes in normal differentiation and cancer remain to be elucidated. With respect to the relationship between methylation and differentiation, studies have hypothesized that two potential mechanisms, loss of cell fate-determining transcription factors by methylation and functional inactivation of corresponding genomic-binding sites by DNA methylation, can promote cellular differentiation defects, thus enhancing the ability of tumor progenitor cells to transition toward ESCC [24]. Negative associations with other clinical factors may be due to the small sample size of this study. Another reason maybe that the analyzed region is not the key promoter region and do not affect PAX4 significantly. In the future, an intensive study with an increased number of patients included is needed to investigate the clinico-epigenetic combination.

No significant correlation has been observed between PTPN22 methylation and prognosis. In other malignancies, gene-specific DNA hypermethylation can predict PFS and OS and is always associated with unfavorable clinical outcomes, lower survival rates and aggressive behavior [25–27]. Our results were not as significant as those of previous studies. In previous studies, a qualitative research-methylation specific PCR (MSP) was used, according to which the methylated and unmethylated groups were divided [28, 29]. A significant correlation was more likely to be observed between methylation and clinicopathological features. However, in the current study, a quantitative research was used to investigate the clinical significance of aberrant methylation of PTPN22. The heterogeneity of cancer cells may impair the trend in the methylation change between ANT and tumor tissue [30]. The heterogeneity weakened the clinical significance of the methylation change in the present study. In addition, the included sample size may not be sufficient to observe a significant difference.

Additionally, there were some limitations to the study. No cell culture was conducted to further investigate the correlate on between PTPN22 and PAX4. This exploration is critical for some findings in the present study. What's more, PTPN22 exhibits overexpression in CLL and positively regulates the antiapoptotic AKT kinase, which provides a powerful survival signal to antigen-stimulated CLL cells [8]. However, no significant difference in PTPN22 expression was shown in ESCC. The expression level of PTPN22 in ESCC merits further exploration in a larger sample size.

In conclusion, hypermethylation of the PTPN22 gene was observed in ESCC. Distinct methylation changes occurred more frequently in non-smoking and N_0_ stage patients. PTPN22 expression was inversely correlated with lymph node metastasis and TNM stage. Lower expression of PTPN22 was associated with poor overall survival. PTPN22 expression may act as a predictor of tumor grade.

## MATERIALS AND METHODS

### Patients and samples

Human primary ESCC and corresponding (5 cm from the tumor) adjacent normal tissue (ANT) were collected from 121 patients who were diagnosed and treated at Fudan University Cancer Hospital (Shanghai, China) from September 2007 to June 2011. The clinicopathological features of the patients are summarized in Table 4. The tissue samples were snap frozen in liquid nitrogen immediately after surgical resection and then were stored at -80°C until DNA and RNA were extracted. The pathological features were evaluated by independent pathologists according to the TNM staging system of the American Joint Committee on Cancer (AJCC 7th edition). Clinical, pathological, and follow-up data were stored in a database in accordance with hospital privacy rules.

**Table 4:** Clinicopathological characteristics of patients

Characters	Type	NO.
Sex	Female	10 (8.3%)
	Male	111 (91.7%)
Alcohol habit (≥50g/day)	No	68 (56.2%)
	Yes	53 (43.8%)
Smoking	No	41 (33.9%)
	Yes	80 (66.1%)
Family History	No	110 (90.9%)
	Yes	11 (9.1%)
Age (mean ± SD)	57.8±6.4	
Diameter of tumor (mean ± SD)	3.03±2.26	
Tumor differentiation status	Poor	13 (10.6%)
	Moderate	68 (55.3%)
	Well	40 (32.5%)
Lesion location	Upper thoracic	44 (36.4%)
	Middle thoracic	46 (38.0%)
	Low thoracic	31 (25.6%)
T stage	T1	7 (5.8%)
	T2	20 (16.5%)
	T3	94 (77.7%)
N stage	N0	57 (47.1%)
	N1	39 (32.2%)
	N2	16 (13.2%)
	N3	9 (7.4%)
TNM stage	I	7 (5.8%)
	II	54 (44.6%)
	III	60 (49.6%)

All patients were followed-up after primary treatment at intervals increasing from 3 months to 1 year until death or the end of the study. The follow-up period ranged from 2 months to 6.1 years (median: 1.7 years) for esophageal cancer patients. Routine examinations, including systemic review, tumor marker testing, endoscopic examination, chest X-ray, and computed tomography, were performed to evaluate the outcome of the disease, which was classified as disease-free, relapse, or death according to the WHO criteria for clinical response. The study protocol was approved by the ethics committee of Fudan University Cancer Center and informed consent was obtained from all participants.

### DNA preparation and bisulfite conversion

Genomic DNA was isolated from ≥25 mg of tissue (tumor/normal) using the QIAamp DNA Mini Kit following the standard protocol (QIAGEN, Hilden, Germany). DNA concentrations were determined using the Thermo NanoDrop2000 system (Thermo, Wilmington, USA). The purity of DNA was verified by monitoring the ratio of absorbance at 260 nm to that at 280 nm, which was in the range of 1.8–2.0. Briefly, 400–500 ng of genomic DNA was chemically modified with sodium bisulfite using the EpiTect Bisulfite kit (QIAGEN, Hilden, Germany) according to the manufacturer's protocol.

### MassARRAY quantitative methylation analysis

The sodium bisulfite-treated genomic DNA was amplified by PCR, and the PCR products were confirmed by gel electrophoresis. The samples were considered positive only if the selected band was comparable to 400- to 500-bp standards. The primer sets used for PCR were as follows: 5′-GGTTGATTAGTTTGAGTTTTTTTGG-3′ (forward primer) and 5′-ACAACAACTCTATCTCAATTCACTACAA-3′ (reverse primer). Only qualified samples were permitted for use in subsequent procedures, including the SAP cleanup, T Cleavage and Clean Resin steps.

The products were then transferred to SpectroCHIP® Arrays, and methylation of the selected region in PTPN22 was detected by MassARRAY spectrometry, an efficient EpiTYPER platform for high throughput analysis of DNA methylation patterns. The gene mass spectrogram was drawn using matrix-assisted laser desorption ionization time-of-flight mass spectrometry (MALDI-TOF MS). The quantitative methylation level of each CpG site or aggregates of multiple CpG sites were analyzed using MassARRAY Analyzer 4 (Sequenom, USA).

### RNA isolation and real-time PCR

Total RNA was isolated using Trizol reagent (Invitrogen, USA) according to the manufacturer's instructions. Agarose gel (1%) electrophoresis and spectrophotometric analysis (OD260:280 nm ratio) were used to evaluate RNA quality. First-strand cDNA was synthesized according to the manufacturer's instructions; 5 μg of total RNA was used to synthesize first-strand cDNA with random six-mer primers using a Superscript III-reverse transcriptase kit (Invitrogen, USA). Following first-strand synthesis, the reaction mixture was diluted to 100 μl with water. Subsequently, 2.5 μl of diluted cDNA mixture was used for PCR amplification in a final 20-μl reaction volume. FastStart Universal SYBR Green Master Mix Rox (Roche, Germany) was used as an amplification reaction mixture according to the manufacturer's instructions. Real-time Quantitative PCR was performed in an ABI VIIA^TM^ 7 Thermal Cycler (Life Technologies, Carlsbad, California, USA). The glyceraldehyde-3-phosphate dehydrogenase (GAPDH) gene was used as an internal control. The following oligonucleotide primers were used: PTPN22-FP, 5′-AGGCAGACAAAACCTATCCTACA-3′; PTPN22-RP, 5′-TGGGTGGCAATATAAGCCTTG-3′; GAPDH-FP, 5′-ACAACTTTGGTATCGTGGAAGG-3′; and GAPDH-RP, 5′-GCCATCACGCCACAGTTTC-3′. All samples were run in triplicate to capture intra-assay variability. The expression level of PTPN22 was analyzed using the 2^−ΔΔCT^ method, where ΔCt = Ct (PTPN22)-Ct (GAPDH) and ΔΔCt = ΔCt (Tumor)−ΔCt (ANT). The expression level in control ANT was set as 1, and the 2^−ΔΔCT^ value was the relative expression level in tumor tissue.

### Statistical analysis

The total methylation differences or individual CpG site methylation differences between the tumor and ANT tissues were analyzed by paired two-tailed Student's t-test. Associations between participant characteristics and methylation differences were assessed using an independent sample t-test and one-way ANOVA. The endpoints were death and disease progression. Overall survival (OS) was calculated from the date of surgery to the date of death or last contact on or before June 2011. Progression-free survival (PFS) was calculated from the date of surgery to the presentation of clinical or pathological evidence of disease recurrence or the last contact on or before June 2011. The Kaplan–Meier method was used for univariate survival analysis, and the log-rank test was used to assess the difference between survival curves. Cox's proportional hazards analysis was used to estimate the prognostic effects of various variables. A *P* value <0.05 was considered to be statistically significant. These statistical calculations were performed using SPSS version 20.0.

## SUPPLEMENTARY MATERIALS FIGURE


