# Supplementary material for: Methylation and expression of PTPN22 in esophageal squamous cell carcinoma
Source: Oncotarget. 2016 Aug 24;7(39):64043–52. doi: 10.18632/oncotarget.11581 (PMC5325424; doi:10.18632/oncotarget.11581)
Supplement: Supplementary file 1 [file oncotarget-07-64043-s001.pdf]

Methylation and expression of PTPN22 in esophageal squamous cell carcinoma

SUPPLEMENTARY FIGURE

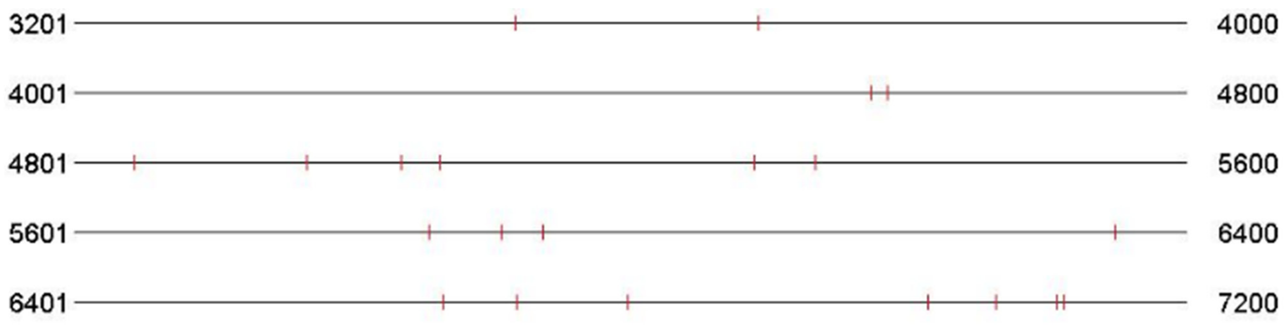

Supplementary Figure S1: Schematic diagram of the CpG sites in PTPN22. The transcriptional start siteis located at 5,000bp.
